# Supplementary material for: Conceptualizing bias in EHR data: A case study in performance disparities by demographic subgroups for a pediatric obesity incidence classifier
Source: PLOS Digit Health. 2024 Oct 23;3(10):e0000642. doi: 10.1371/journal.pdig.0000642 (PMC11498669; doi:10.1371/journal.pdig.0000642)
Supplement: S1 Table — (DOCX) [file pdig.0000642.s001.docx]

**S1 Table.** Inclusion and Exclusion Criteria for Study Population

| Inclusion Criteria: | Exclusion Criteria |
| --- | --- |
| - Case patients had at least one measurement of obesity (BMI z-score at or above the 95^th^ percentile for age and sex) at a CHOP primary care visit between January 1, 2009 and December 31, 2016 - All patients are 2-18 years old during index visit - BMI measurement in the pre-index and index visit for all study population patients - Non-obese BMI measurement in the pre-index visit and obese BMI in the index visit for case patients - Non-obese BMI measurement in both the pre- and index visits for control patients - Record of insurance information within 2 years of the matched index visit for cases and controls | - Missing BMI measurements in pre-index and index visits - BMI measurements were not in the healthy range for BMI measurements for control patients - BMI was not in the healthy range for case patients in the pre-index visit - Matched case or control patient did not meet the inclusion criteria |
