# Supplementary material for: Conceptualizing bias in EHR data: A case study in performance disparities by demographic subgroups for a pediatric obesity incidence classifier
Source: PLOS Digit Health. 2024 Oct 23;3(10):e0000642. doi: 10.1371/journal.pdig.0000642 (PMC11498669; doi:10.1371/journal.pdig.0000642)
Supplement: S2 Table — (DOCX) [file pdig.0000642.s002.docx]

### **S2 Table.** Temporal Diagnoses Included as Machine Learning Model Features

| **Diagnoses** |
| --- |
| *Pre-Index Visit Diagnoses*  1-Chronic pharyngitis and tonsillitis  1-Deafness, hearing loss  1-Respiratory signs and symptoms  1-Sleep Apnea  1-Sleep Problems  1-Dermatitis and eczema  1-Seizure Disorder  1-Asthma w/o Status Asthmaticus  1-Constipation  1-Urinary Symptoms  1-Autism Spectrum Disorder  1-Deafness, hearing loss  1-Fever  1-Gasteroenteritis  1-Headaches  1-Nausea, vomiting |
| *Index Visit Diagnoses*  2-Chronic pharyngitis and tonsillitis  2-Respiratory signs and symptoms  2-Sleep Apnea  2-Sleep Problems  2-Allergic Rhinitis  2-Dermatitis and eczema  2-Developmental disorder  2-Neurologic signs and symptoms  2-Seizure Disorder  2-Constipation  2-Urinary Symptoms  2-Allergic Rhinitis  2-Asthma w/o Status Asthmaticus  2-Dermatitis and eczema  2-Autism Spectrum Disorder  2-Developmental disorder  2-Neurologic signs and symptoms  2-Headaches |
