# Supplementary material for: Conceptualizing bias in EHR data: A case study in performance disparities by demographic subgroups for a pediatric obesity incidence classifier
Source: PLOS Digit Health. 2024 Oct 23;3(10):e0000642. doi: 10.1371/journal.pdig.0000642 (PMC11498669; doi:10.1371/journal.pdig.0000642)
Supplement: S3 Table — (DOCX) [file pdig.0000642.s003.docx]

### **S3 Table.** Demographic Characteristics of Test and Training Set Patient Populations

|  | | Training Set Patient Population | | | Test Set Patient Population | | |
| --- | --- | --- | --- | --- | --- | --- | --- |
|  | % (SD) | | % (SD) | | |  |  |
|  |  | | |  |  |  |  |
| *Sex* |  | | |  |  |  |  |
| Male | | 55.4% (0.3) | | 55.4% (0.9) | | |  |
| Female | | 44.6% (0.3) | | 44.6% (0.9) | | |  |
| *Race/ethnicity* | |  | |  | | |  |
| Asian | | 3.0% (0.1) | | 3.0% (0.3) | | |  |
| Black/African American | | 25.3% (0.2) | | 25.3% (0.7) | | |  |
| White | | 60.1% (0.3) | | 60.1% (0.9) | | |  |
| Hispanic | | 3.5% (0.1) | | 3.5% (0.3) | | |  |
| Multiple Race | | 1.3% (0.1) | | 1.0% (0.2) | | |  |
| Heterogeneous Other | | <1% | | <1% | | |  |
| Unknown | | 6.7% (0.2) | | 6.7% (0.3) | | |  |
|  | |  | |  | | |  |
| *Medicaid Enrollment* | |  | |  | | |  |
| Medicaid/CHIP | | 32.1% (0.3) | | 32.1% (0.9) | | |  |
| *Age at index visit* | |  | |  | | |  |
| 2-4 years | | 25.4% (0.2) | | 25.4% (0.8) | | |  |
| 5-11 years | | 39.7% (0.3) | | 39.7% (0.8) | | |  |
| 12-18 years | | 34.9% (0.3) | | 35.0% (0.8) | | |  |
