# Supplementary material for: Conceptualizing bias in EHR data: A case study in performance disparities by demographic subgroups for a pediatric obesity incidence classifier
Source: PLOS Digit Health. 2024 Oct 23;3(10):e0000642. doi: 10.1371/journal.pdig.0000642 (PMC11498669; doi:10.1371/journal.pdig.0000642)
Supplement: S4 Table — (DOCX) [file pdig.0000642.s004.docx]

**S4 Table. Pairwise model performance comparison.** ANOVA results comparing model performance overall and for demographic subgroups. P-value <0.05 indicates a statistically significant difference in model performance.

|  | *RF* | *LR* | *NN* |
| --- | --- | --- | --- |
| Overall Performance |  |  |  |
| *GBT* | <0.01 | 0.02 | <0.01 |
| *RF* |  | <0.01 | <0.01 |
| *LR* |  |  | <0.01 |
|  |  |  |  |
| Gender |  |  |  |
| Male |  |  |  |
| *GBT* | <0.01 | 0.46 | <0.01 |
| *RF* |  | <0.01 | <0.01 |
| *LR* |  |  | <0.01 |
|  |  |  |  |
| Female |  |  |  |
| *GBT* | <0.01 | 0.63 | <0.01 |
| *RF* |  | <0.01 | 0.04 |
| *LR* |  |  | <0.01 |
|  |  |  |  |
| race/  ethnicity |  |  |  |
| Asian |  |  |  |
| *GBT* | 0.11 | 0.91 | <0.01 |
| *RF* |  | 0.16 | <0.01 |
| *LR* |  |  | <0.01 |
|  |  |  |  |
| African american |  |  |  |
| *GBT* | <0.01 | <0.01 | <0.01 |
| *RF* |  | <0.01 | <0.01 |
| *LR* |  |  | 0.61 |
|  |  |  |  |
| white |  |  |  |
| *GBT* | <0.01 | <0.01 | <0.01 |
| *RF* |  | <0.01 | <0.01 |
| *LR* |  |  | <0.01 |
|  |  |  |  |
| Hispanic |  |  |  |
| *GBT* | 0.31 | <0.01 | <0.01 |
| *RF* |  | <0.01 | 0.02 |
| *LR* |  |  | <0.01 |
|  |  |  |  |
| multiple |  |  |  |
| *GBT* | 0.51 | <0.01 | 0.50 |
| *RF* |  | <0.01 | 0.17 |
| *LR* |  |  | 0.01 |
|  |  |  |  |
| Unknown |  |  |  |
| *GBT* | 0.37 | 0.30 | <0.01 |
| *RF* |  | 0.92 | <0.01 |
| *LR* |  |  | <0.01 |
|  |  |  |  |
| medicaid |  |  |  |
| *GBT* | <0.01 | <0.01 | <0.01 |
| *RF* |  | <0.01 | 0.85 |
| *LR* |  |  | <0.01 |
|  |  |  |  |
| Age |  |  |  |
| 2-4 Years |  |  |  |
| *GBT* | <0.01 | 0.80 | <0.01 |
| *RF* |  | <0.01 | <0.01 |
| *LR* |  |  | <0.01 |
|  |  |  |  |
| 5-11 Years |  |  |  |
| *GBT* | <0.01 | 0.16 | <0.01 |
| *RF* |  | <0.01 | 0.30 |
| *LR* |  |  | <0.01 |
|  |  |  |  |
| 12-18 Years |  |  |  |
| *GBT* | <0.01 | <0.01 | <0.01 |
| *RF* |  | <0.01 | <0.01 |
| *LR* |  |  | <0.01 |
