# Supplementary material for: Conceptualizing bias in EHR data: A case study in performance disparities by demographic subgroups for a pediatric obesity incidence classifier
Source: PLOS Digit Health. 2024 Oct 23;3(10):e0000642. doi: 10.1371/journal.pdig.0000642 (PMC11498669; doi:10.1371/journal.pdig.0000642)
Supplement: S5 Table — (DOCX) [file pdig.0000642.s005.docx]

**S5 Table. Intra-model subgroup performance comparison.** ANOVA results comparing model performance for demographic subgroups for each model. P-value <0.05 indicates a statistically significant difference in model performance

|  | *GBT* | *RF* | *LR* | *NN* |
| --- | --- | --- | --- | --- |
| Sex | 0.07 | 0.37 | 0.04 | <0.01 |
|  |  |  |  |  |
| Race/  Ethnicity |  |  |  |  |
| Asian | <0.01 | 0.02 | 0.01 | <0.01 |
| African American | <0.01 | <0.01 | <0.01 | <0.01 |
| White | <0.01 | <0.01 | <0.01 | <0.01 |
| Hispanic | <0.01 | <0.01 | 0.16 | <0.01 |
| Multiple | <0.01 | <0.01 | 0.38 | 0.08 |
| unknown | <0.01 | <0.01 | <0.01 | <0.01 |
|  |  |  |  |  |
| medicaid | <0.01 | <0.01 | <0.01 | <0.01 |
|  |  |  |  |  |
| Age |  |  |  |  |
| 2-4 years | <0.01 | 0.78 | <0.01 | <0.01 |
| 5-11 years | <0.01 | <0.01 | <0.01 | <0.01 |
| 12-18 years | <0.01 | <0.01 | <0.01 | <0.01 |
